# Emergence of SARS-CoV-2 Omicron lineages BA.4 and BA.5 in South Africa

**DOI:** 10.1038/s41591-022-01911-2

**Published:** 2022-06-27

**Authors:** Houriiyah Tegally, Monika Moir, Josie Everatt, Marta Giovanetti, Cathrine Scheepers, Eduan Wilkinson, Kathleen Subramoney, Zinhle Makatini, Sikhulile Moyo, Daniel G. Amoako, Cheryl Baxter, Christian L. Althaus, Ugochukwu J. Anyaneji, Dikeledi Kekana, Raquel Viana, Jennifer Giandhari, Richard J. Lessells, Tongai Maponga, Dorcas Maruapula, Wonderful Choga, Mogomotsi Matshaba, Mpaphi B. Mbulawa, Nokukhanya Msomi, NGS-SA consortium, Armand Phillip Bester, Mathilda Claassen, Deelan Doolabh, Innocent Mudau, Nokuzola Mbhele, Susan Engelbrecht, Dominique Goedhals, Diana Hardie, Nei-Yuan Hsiao, Arash Iranzadeh, Arshad Ismail, Rageema Joseph, Arisha Maharaj, Boitshoko Mahlangu, Kamela Mahlakwane, Ashlyn Davis, Gert Marais, Koleka Mlisana, Anele Mnguni, Thabo Mohale, Gerald Motsatsi, Peter Mwangi, Noxolo Ntuli, Martin Nyaga, Luicer Olubayo, Botshelo Radibe, Yajna Ramphal, Upasana Ramphal, Wilhelmina Strasheim, Naume Tebeila, Stephanie van Wyk, Shannon Wilson, Alexander G. Lucaci, Steven Weaver, Akhil Maharaj, Yusasha Pillay, Michaela Davids, Adriano Mendes, Simnikiwe Mayaphi, Yeshnee Naidoo, Sureshnee Pillay, Tomasz Janusz Sanko, James E. San, Lesley Scott, Lavanya Singh, Nonkululeko A. Magini, Pamela Smith-Lawrence, Wendy Stevens, Graeme Dor, Derek Tshiabuila, Nicole Wolter, Wolfgang Preiser, Florette K. Treurnicht, Marietjie Venter, Georginah Chiloane, Caitlyn McIntyre, Aine O’Toole, Christopher Ruis, Thomas P. Peacock, Cornelius Roemer, Sergei L. Kosakovsky Pond, Carolyn Williamson, Oliver G. Pybus, Jinal N. Bhiman, Allison Glass, Darren P. Martin, Ben Jackson, Andrew Rambaut, Oluwakemi Laguda-Akingba, Simani Gaseitsiwe, Anne von Gottberg, Tulio de Oliveira

**Affiliations:** 1 Centre for Epidemic Response and Innovation (CERI), School of Data Science and Computational Thinking, Stellenbosch University, Stellenbosch, South Africa; 2 KwaZulu-Natal Research Innovation and Sequencing Platform (KRISP), Nelson R. Mandela School of Medicine, University of KwaZulu-Natal, Durban, South Africa; 3 National Institute for Communicable Diseases (NICD) of the National Health Laboratory Service (NHLS), Johannesburg, South Africa; 4 Laboratorio de Flavivirus, Fundacao Oswaldo Cruz, Rio de Janeiro, Brazil; 5 Department of Science and Technology for Humans and the Environment, University of Campus Bio-Medico di Roma, Rome, Italy; 6 Laboratório de Genética Celular e Molecular, Universidade Federal de Minas Gerais, Belo Horizonte, Brazil; 7 South African Medical Research Council Antibody Immunity Research Unit, School of Pathology, Faculty of Health Sciences, University of the Witwatersrand, Johannesburg, South Africa; 8 Department of Virology, Charlotte Maxeke Johannesburg Academic Hospital, Johannesburg, South Africa; 9 School of Pathology, Faculty of Health Sciences, University of the Witwatersrand, Johannesburg, South Africa; 10 Botswana Harvard AIDS Institute Partnership, Botswana Harvard HIV Reference Laboratory, Gaborone, Botswana; 11 Harvard T.H. Chan School of Public Health, Boston, MA USA; 12 Botswana Presidential COVID-19 Taskforce, Gaborone, Botswana; 13 Institute of Social and Preventive Medicine, University of Bern, Bern, Switzerland; 14 Lancet Laboratories, Johannesburg, South Africa; 15 Division of Medical Virology, Faculty of Medicine and Health Sciences, Stellenbosch University, Cape Town, South Africa; 16 National Health Laboratory, Health Services Management, Ministry of Health and Wellness, Gaborone, Botswana; 17 Discipline of Virology, School of Laboratory Medicine and Medical Sciences and National Health Laboratory Service (NHLS), University of KwaZulu-Natal, Durban, South Africa; 18 Department of Molecular Medicine and Haematology, Faculty of Health Science, School of Pathology, University of the Witwatersrand, Johannesburg, South Africa; 19 Health Services Management, Ministry of Health and Wellness, Gaborone, Botswana; 20 National Priority Program of the National Health Laboratory Service, Johannesburg, South Africa; 21 Zoonotic Arbo and Respiratory Virus Program, Centre for Viral Zoonoses, Department of Medical Virology, University of Pretoria, Pretoria, South Africa; 22 Institute of Evolutionary Biology, University of Edinburgh, Edinburgh, UK; 23 Department of Medicine, University of Cambridge, Cambridge, UK; 24 Department of Infectious Disease, Imperial College London, London, UK; 25 Biozentrum, University of Basel, Basel, Switzerland; 26 Institute for Genomics and Evolutionary Medicine, Department of Biology, Temple University, Philadelphia, PA USA; 27 Division of Medical Virology, Faculty of Health Sciences, University of Cape Town, Cape Town, South Africa; 28 Division of Virology, NHLS Groote Schuur Laboratory, Cape Town, South Africa; 29 Wellcome Centre for Infectious Diseases Research in Africa (CIDRI-Africa), Cape Town, South Africa; 30 Institute of Infectious Disease and Molecular Medicine, University of Cape Town, Cape Town, South Africa; 31 Department of Zoology, University of Oxford, Oxford, UK; 32 NHLS Port Elizabeth Laboratory, Port Elizabeth, South Africa; 33 Faculty of Health Sciences, Walter Sisulu University, Eastern Cape, South Africa; 34 Division of Medical Microbiology, Department of Pathology, Faculty of Health Sciences, University of Cape Town, Cape Town, South Africa; 35 Department of Global Health, University of Washington, Seattle, WA USA; 36 Division of Virology, National Health Laboratory Service, Bloemfontein, South Africa; 37 Division of Virology, University of the Free State, Bloemfontein, South Africa; 38 PathCare Vermaak, Pretoria, South Africa; 39 Division of Computational Biology, Faculty of Health Sciences, University of Cape Town, Cape Town, South Africa; 40 NHLS Tygerberg Laboratory, Cape Town, South Africa; 41 National Health Laboratory Service (NHLS), Johannesburg, South Africa; 42 Centre for the AIDS Programme of Research in South Africa (CAPRISA), Durban, South Africa; 43 Next Generation Sequencing Unit, Division of Virology, Faculty of Health Sciences, University of the Free State, Bloemfontein, South Africa; 44 Department of Medical Virology, University of Pretoria, Pretoria, South Africa

**Keywords:** Epidemiology, Phylogeny

## Abstract

Three lineages (BA.1, BA.2 and BA.3) of the severe acute respiratory syndrome coronavirus 2 (SARS-CoV-2) Omicron variant of concern predominantly drove South Africa’s fourth Coronavirus Disease 2019 (COVID-19) wave. We have now identified two new lineages, BA.4 and BA.5, responsible for a fifth wave of infections. The spike proteins of BA.4 and BA.5 are identical, and similar to BA.2 except for the addition of 69–70 deletion (present in the Alpha variant and the BA.1 lineage), L452R (present in the Delta variant), F486V and the wild-type amino acid at Q493. The two lineages differ only outside of the spike region. The 69–70 deletion in spike allows these lineages to be identified by the proxy marker of S-gene target failure, on the background of variants not possessing this feature. BA.4 and BA.5 have rapidly replaced BA.2, reaching more than 50% of sequenced cases in South Africa by the first week of April 2022. Using a multinomial logistic regression model, we estimated growth advantages for BA.4 and BA.5 of 0.08 (95% confidence interval (CI): 0.08–0.09) and 0.10 (95% CI: 0.09–0.11) per day, respectively, over BA.2 in South Africa. The continued discovery of genetically diverse Omicron lineages points to the hypothesis that a discrete reservoir, such as human chronic infections and/or animal hosts, is potentially contributing to further evolution and dispersal of the virus.

## Abstract (web summary)

Genomic characterization of the SARS-CoV-2 Omicron lineages BA.4 and BA.5, responsible for the fifth COVID-19 pandemic wave in South Africa, shows continued viral diversification and provides insights into the potential mechanisms underlying the ability of the new lineages to outcompete their predecessors.

## Main

Within days of being discovered in South Africa and Botswana, on 26 November 2021, the Omicron variant of SARS-CoV-2 was designated as a variant of concern by the World Health Organization^1^. Initially, Omicron was comprised of three sister lineages: BA.1, BA.2 and BA.3. BA.1 caused most of the infections in South Africa’s fourth epidemic wave. However, as that wave receded in mid-January 2022, BA.2 became the dominant South African lineage. Despite being associated with a modest prolongation of the fourth wave, the displacement of BA.1 by BA.2 in South Africa was not associated with a substantial resurgence in cases, hospital admissions or deaths. This pattern was not consistent worldwide, however, and, in some countries, BA.2 was responsible for a greater share of cases, hospitalizations and deaths during the Omicron wave^2–4^.

**Fig. 1:**
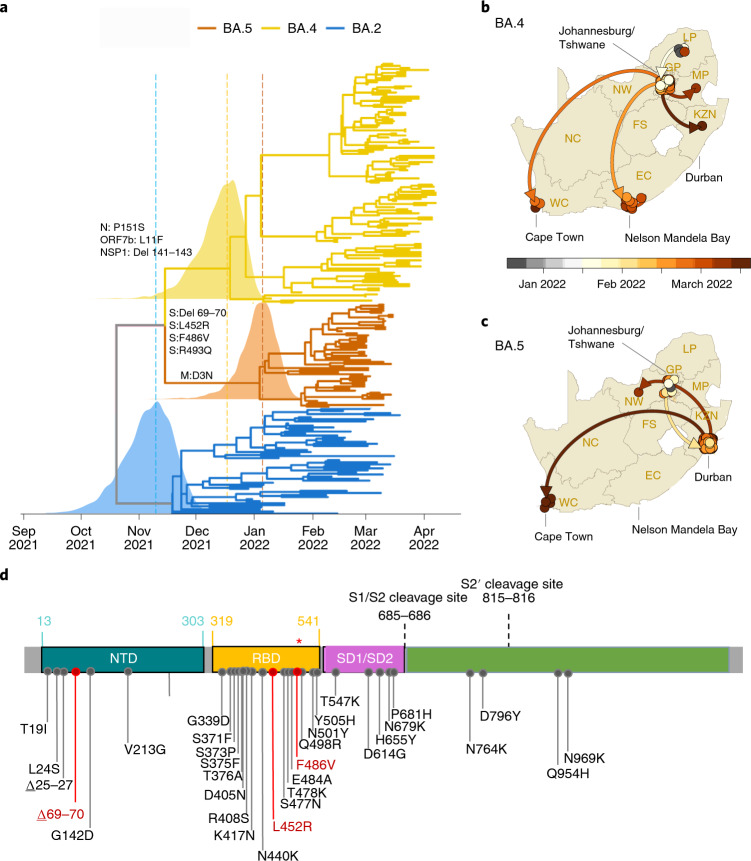
Molecular Evolution and Profile of BA.4 and BA.5 lineages. **a**, Time-resolved maximum clade credibility phylogeny of the BA.2, BA.4 and BA.5 lineages (*n* = 221, sampled between 29 December 2021 and 7 April 2022). Mutations that characterize the lineages are indicated on the branch at which each first emerged. The posterior distribution of the TMRCA is also shown for BA.2, BA.4 and BA.5. **b**, Spatiotemporal reconstruction of the spread of the BA.4 lineage in South Africa. **c**, Spatiotemporal reconstruction of the spread of the BA.5 lineage in South Africa. In **b** and **c**, circles represent nodes of the maximum clade credibility phylogeny, colored according to their inferred time of occurrence (scale shown). EC, Eastern Cape; FS, Free State; GP, Gauteng; KZN, KwaZulu-Natal; LP, Limpopo; MP, Mpumalanga; NC, Northern Cape; NW, North West; WC, Western Cape. Solid curved lines denote the links between nodes, and the directionality of movement is indicated (anti-clockwise along the curve). **d**, Amino acid mutations in the spike gene of the BA.4 and BA.5 lineages. Mutations that differ from BA.2 are denoted in red, including the wild-type amino acid at position Q493 (denoted by the red asterisk (*)). NTD, N-terminal domain; SD1, subdomain 1; SD2, subdomain 2.

**Extended Data Fig. 1:**
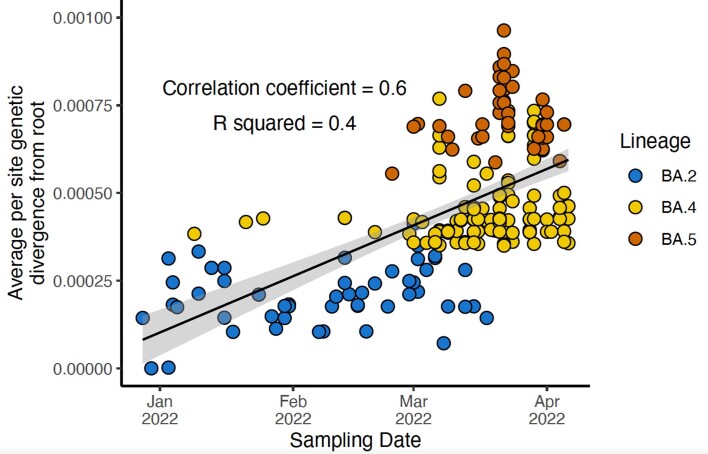
Molecular clock signal of the dataset of BA.2, BA.4 and BA.5 lineages used in the Bayesian analysis. Root-to-tip regression obtained from TempEst analysis for the sampled cluster of BA.2, BA.4 and BA.5, showing a relatively strong clock-like behaviour (correlation coefficient = 0.6, R2 = 0.4) The regression line (representing the estimated mean evolutionary rate) is shown with error buffers (shaded area) that represent 90% confidence intervals.

We recently identified two new Omicron lineages that have been designated BA.4 and BA.5 by the Pango Network and pango-designation version 1.3, a system of naming and classifying SARS-CoV-2 lineages (Fig. 1a)^5,6^. Bayesian phylogenetic methods revealed that BA.4 and BA.5 are distinct from the other Omicron lineages (molecular clock signal: correlation coefficient = 0.6, R^2^ = 0.4; Extended Data Fig. 1). BA.4 and BA.5 are estimated to have originated in mid-December 2021 (95% highest posterior density (HPD): 25 November 2021 to 1 January 2022) and early January 2022 (HPD: 10 December 2021 to 6 February 2022), respectively (Fig. 1a). The most recent common ancestor of BA.4 and BA.5 is estimated to have originated in mid-November 2021 (HPD: 29 September 2021 to 6 December 2021) (Fig. 1a), coinciding with the emergence of the other lineages—for example, BA.2 in early November 2021 (HPD: 9 October 2021 to 29 November 2021). Phylogeographic analysis suggests early dispersal of BA.4 from Limpopo to Gauteng, with later spread to other provinces (Fig. 1b), and early dispersal of BA.5 from Gauteng to KwaZulu-Natal, with more limited onward spread to other provinces (Fig. 1c).

**Extended Data Fig. 2:**
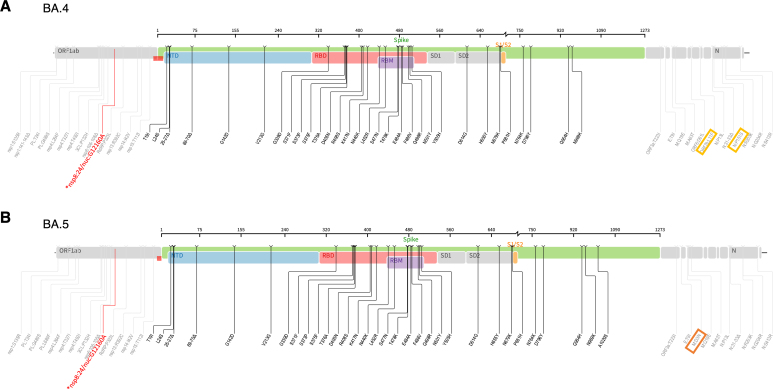
Whole genome mutations present in BA.4 and BA.5 lineages. Differences in BA.4 and BA.5 are highlighted with a rectangle. The synonymous mutations in nsp8 is indicated in red.

BA.4 and BA.5 have identical spike proteins, most similar to BA.2. Relative to BA.2, BA.4 and BA.5 have the additional spike mutations 69–70 deletion, L452R, F486V and wild-type amino acid at position Q493 (Fig. 1d). Outside of spike, BA.4 has additional mutations at ORF7b:L11F and N:P151S and a triple amino acid deletion in NSP1:141–143 deletion, whereas BA.5 has the M:D3N mutation. Relative to BA.2, BA.5 has additional reversions at ORF6:D61 and nucleotide positions 26,858 and 27,259. In addition, BA.4 and BA.5 have a nuc:G12160A synonymous mutation in NSP8 that was present in Epsilon (B.1.429) and has arisen in BA.2 in some locations (Extended Data Fig. 2). BA.4 and BA.5 have identical mutational patterns in the 5′ genome region (from ORF1ab to Envelope) yet exhibit genetic divergence in the 3′ region (from M to the 3′ genome end). This suggests that BA.4 and BA.5 may be related by a recombination event, with breakpoint between the E and M genes, before their emergence into the general population. This scenario is somewhat similar to the relationship between BA.3 and BA.1/BA.2, which also exhibit apparent ancestral recombination^1^. Using the RASCL pipeline^7^ (which employs a battery of tests that analyze ratios of synonymous and non-synonymous substitutions both at individual codon sites and entire protein-coding regions), we found no compelling evidence of imbalances between ratios of synonymous and non-synonymous substitutions such as would be indicative of positive selection (that is, favoring amino acid changes) or negative selection (that is, disfavoring amino acid changes) acting on any of the genes of viruses in either the BA.4 or BA.5 lineages.

It is currently unknown how differences in the mutation profiles of BA.4 and BA.5, relative to BA.2, will affect their phenotypes. Changes at spike amino acids 452, 486 and 493 are likely to influence human angiotensin-converting enzyme-2 (hACE2) and antibody binding. The 452 residue is in immediate proximity to the interaction interface of the hACE2 receptor. The L452R mutation has been associated with an increased affinity for receptor binding with a resultant increased in vitro infectivity^8^. The L452R mutation is also present in the Delta, Kappa and Epsilon variants (and L452Q in Lambda), and mutations at this position have been associated with a reduction in neutralization by monoclonal antibodies (particularly class 2 antibodies) and polyclonal sera^9–11^. Mutations at this position (L452R/M/Q) have also arisen independently in several BA.2 sublineages in different parts of the world, most notably BA.2.12.1 (L452Q), which has become dominant in many parts of the United States. It is, therefore, unclear whether BA.4/BA.5 will become dominant throughout the world or whether there will be a period of co-circulation of several different Omicron lineages.

**Extended Data Fig. 3:**
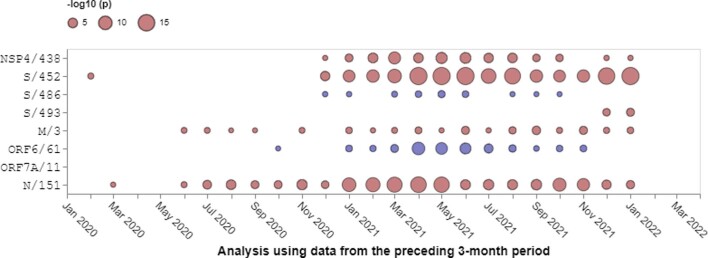
Patterns of natural selection between January 2020 and January 2022 at codon sites differentiating BA.4 and BA.5 from BA.2. All SARS-CoV-2 sequences deposited in GISAID were analyzed with each time-point representing an analysis of all sequences sampled during the preceding three months. Red dots indicate evidence at positive selection and blue spots indicate evidence of negative selection. The sizes of the dots indicate degrees of statistical support for selection signals. Only sequences deposited in GISAID prior to the discovery of BA.4 and BA.5 are considered here.

Before the emergence of BA.4 and BA.5, F486V in the receptor-binding domain (RBD) of spike had been observed in only 54 of 10 million publicly available genome sequences in GISAID (https://cov-spectrum.org/explore/World/AllSamples/AllTimes/variants?aaMutations=S%3AF486V&). Selection analyses focusing on ratios of non-synonymous and synonymous substitution rates at individual codons have indicated that, since December 2020, S:486 has been evolving under strong negative selection favoring the F state at this site (that is, the amino acid that is found in Wuhan-Hu-1) (Extended Data Fig. 3). Although rare, the F486L mutation has been observed in approximately 500 genomes, most commonly in viruses infecting minks and from human cases linked to mink farms. The F486L mutation has been shown to directly enhance entry into cells expressing mink or ferret ACE2 (ref. ^12^). When binding to hACE2, spike amino acid F486 interacts with hACE2 residues L79, M82 and Y83, which collectively comprise a hotspot for ACE2 differences between mammalian species^13^. Mutations at F486 are associated with a reduction in neutralizing activity by class 1 (and some class 2) neutralizing antibodies and by polyclonal sera^9–11^. Deep mutational scanning suggests that F486 is a key site for escape of vaccine-elicited and infection-elicited RBD-targeted antibodies, including those still able to neutralize Omicron/BA.1 (https://jbloomlab.github.io/SARS2_RBD_Ab_escape_maps/escape-calc/)^14^. This suggests that BA.4 and BA.5 may be even better at evading neutralizing antibody responses, including those recently elicited by BA.1 infections. Combined with waning population immunity against infection from the initial Omicron/BA.1 wave, this could create the conditions for a substantial resurgence in infections.

**Fig. 2:**
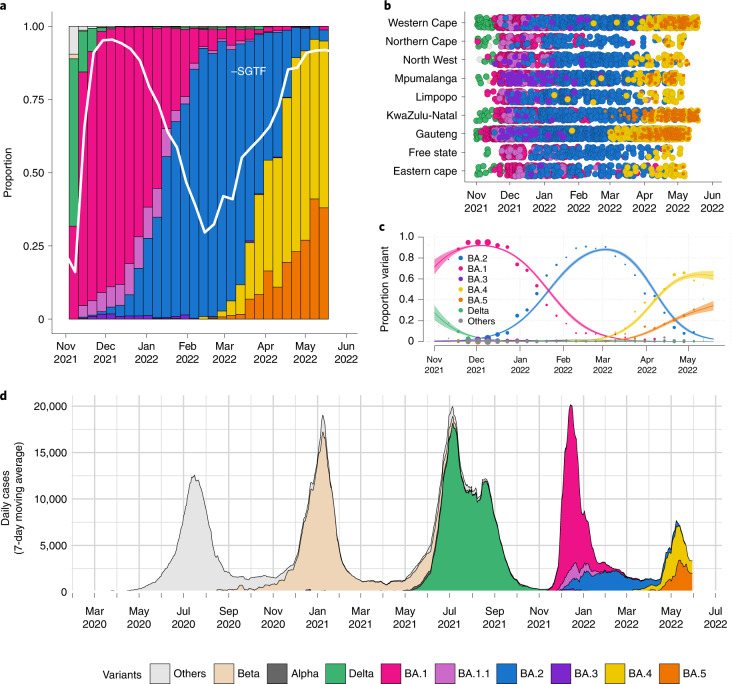
Distribution of SARS-CoV-2 lineages in South Africa. **a**, Changes in the genomic prevalence of Omicron lineages in South Africa from November 2021 (when BA.1 dominated) to May 2022 (when BA.4 and BA.5 were increasing in frequency), superimposed with the proportion of positive TaqPath qPCR tests exhibiting SGTF from November 2021 to May 2022. Estimations of genomic prevalence and SGTF proportions are done from different samples and datasets and presented together here only for illustrative purposes. **b**, The count of Omicron lineage genomes per province of South Africa over November 2021 to May 2022. BA.4 and BA.5 have been detected in all nine provinces. **c**, Modeled linear proportions of the Omicron lineages in South Africa. BA.1 rapidly outcompeted Delta in November 2021 and was then superseded by BA.2 in early 2022. BA.4 and BA.5 appear to be swiftly replacing BA.2 in South Africa. Model fits are based on a multinomial logistic regression, and dot size represents the weekly sample size. The shaded areas correspond to the 95% CIs of the model estimates. **d**, The progression of the 7-day rolling average of daily reported case numbers in South Africa over 2 years of the epidemic (April 2020 to May 2022). Daily cases are colored by the inferred proportion of SARS-CoV-2 variants prevalent at a particular period in the epidemic.

The S:69–70 deletion means that BA.4 and BA.5 can again be presumptively identified (against a background of BA.2 infection) using the proxy marker of S-gene target failure (SGTF) with the TaqPath COVID-19 qPCR assay (Thermo Fisher Scientific). SGTF was successfully used to track the early spread of BA.1 (which also demonstrates SGTF), later also enabling discrimination between BA.1 and BA.2 infections, because BA.2 viruses generally lack the S:69–70 deletion^15^. Recent data from public laboratories in South Africa suggest that the proportion of positive polymerase chain reaction (PCR) tests with SGTF has been increasing since early March, suggesting that BA.4 and BA.5 may be responsible for a growing share of recently confirmed cases (Fig. 2a). To assess the validity of SGTF for identifying BA.4/BA.5, we performed quantitative PCR (qPCR) with the TaqPath assay on 296 unselected samples submitted for sequencing to the KwaZulu-Natal Research Innovation and Sequencing Platform (KRISP) from Gauteng, Eastern Cape and KwaZulu-Natal collected between 6 January and 3 April 2022. Of the 296 samples processed, we had a paired valid qPCR result and sequence for 198. Of the 77 samples with SGTF on qPCR, 66 were BA.4 or BA.5, nine were BA.1 and two were BA.2. No BA.4 and BA.5 genomes were S-gene target positive on qPCR (Extended Data Table 1). These results suggest that SGTF surveillance (where the assay is available) may, for now, be a reasonable proxy to identify BA.4 and BA.5 for countries with a low prevalence of BA.1.

**Extended Data Fig. 4:**
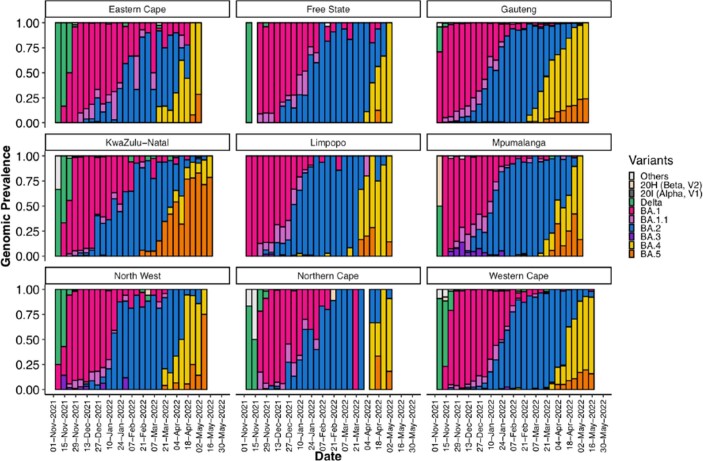
Progression of the weekly genomic prevalence of various variants and lineages in the nine provinces of South Africa from November 2021 to May 2022.

At the time of this writing, we have confirmed BA.4 and/or BA.5 in all nine provinces in South Africa (Eastern Cape, Gauteng, KwaZulu-Natal, Limpopo, Mpumalanga, North West, Northern Cape, Free State and Western Cape) in samples collected between 10 January 2022 and 19 May 2022 (Fig. 2b). In the two most populous provinces in South Africa—Gauteng and KwaZulu-Natal—BA.4 and BA.5 rapidly replaced BA.2 and were responsible for approximately 90% of sequenced cases by the week starting 18 April 2022 (Extended Data Fig. 4). These estimates are based on unselected sampling for genomic surveillance (samples not selected based on SGTF or genotyping). The data suggest geographic heterogeneity in the distribution of these two new lineages, with growth predominantly of BA.4 in Gauteng and BA.5 in KwaZulu-Natal (Extended Data Fig. 4). Internationally, by the end of May 2022, BA.4 and BA.5 had also been detected and were rising in prevalence in several countries: in neighboring Botswana (estimated prevalence 60%), in Europe (Portugal, Spain and Austria) and in the United States.

We estimated that Omicron BA.4 and BA.5 had a daily growth advantage of 0.08 (95% CI: 0.08–0.09) and 0.10 (95% CI: 0.09–0.11), respectively, relative to BA.2 in South Africa in May 2022 (Fig. 2f). These estimates are similar to the estimated daily growth advantage of 0.07 (95% CI: 0.07–0.06) of BA.2 over BA.1 in February 2022 (Fig. 2c). The BA.4 and BA.5 lineages also show a growth advantage against non-Omicron lineages, although these are minimally circulating in the discussed timeframe (Extended Data Table 2). The growth advantage of Omicron BA.4 and BA.5 could be mediated by (1) an increase in its intrinsic transmissibility relative to other variants; (2) an increase relative to other variants in its capacity to infect, and be transmitted from, previously infected and vaccinated individuals; or (3) both. The estimated time to most recent common ancestor for both BA.4 and BA.5 (mid-November 2021, similar to that for BA.1 and BA.2) argues against the first option because that suggests both lineages would have been circulating throughout the period dominated by BA.1 and then BA.2 without exhibiting a transmission advantage. The observation that both BA.4 and BA.5 (and many lineages within them) have recently started to grow in frequency suggests that the growth advantage is recent and uniform across these lineages. It is estimated that almost all of the South African population has some degree of immunity to SARS-CoV-2, provided by a complex mixture of vaccination and prior infections with wild-type, Beta, Delta and Omicron (particularly BA.1) (Fig. 2d)^16,17^. Given that the transmission advantage becomes apparent approximately 4 months from the start of the Omicron wave, it is plausible that waning immunity (particularly that acquired from BA.1 infection) is an important contributory factor. This would also suggest that the effects of these different Omicron lineages may differ by location, depending on the immune landscape and, particularly, the patterns of exposure to BA.1 and BA.2.

At the time of this writing, a wave of infections caused by the BA.4 and BA.5 lineages was ending in South Africa (Fig. 2d). This wave was characterized by a peak in test positivity rate of ~24%, lower than during the Omicron BA.1 wave (~34%), and, because of high population immunity, much lower hospital admissions and deaths than previously recorded during waves of infection in South Africa. It is worth noting that recorded death metrics were further decoupled from cases and hospitalizations compared to the BA.1 wave. The ability of the BA.4 and BA.5 lineages to drive a new wave of infections can potentially be explained by their ability to evade immunity induced by the BA.1 lineage roughly 3 months after infection^18^. The fifth wave in South Africa, driven by BA.4 and BA.5, occurred around 4 months after the fourth wave, driven by BA.1. At the time of writing this report, Botswana was experiencing a rapid rise in cases driven by BA.4 and BA.5, with 19 of 24 health districts experiencing resurgence in cases. To note, Botswana’s fourth wave was driven by BA.1, followed by BA.2 lasting about 3.5 months, and the country’s fifth wave is occurring approximately 2 months after the fourth wave.

This study has several limitations. First, the estimated growth advantage of the BA.4 and BA.5 lineages could be biased due to stochastic effects (such as superspreading) in a low-incidence setting at the start of a wave, which can lead to overestimates of the growth advantage. Second, reliable estimates of the level of population immunity against BA.1 in South Africa are not yet available, making it difficult to precisely estimate transmissibility or immune evasion of the new lineages. There also remains some uncertainty about the origin of the different Omicron lineages, and phylogenetic inference is limited by the relatively low sampling coverage in our genomic surveillance (<1% of confirmed cases in South Africa). Furthermore, the lack of sampling of an ancestor of the different Omicron lineages complicates phylogenetic placements. Although the Bayesian phylogenetic methods employed here suggest that BA.4 and BA.5 are independent lineages that originated around the same time as BA.1–BA.3, maximum likelihood estimations suggest that they could have descended from BA.2. Further sequencing (particularly samples from Gauteng and neighboring provinces) may help to provide more clarity.

The continued discovery of genetically diverse Omicron lineages shifts the level of support for hypotheses regarding their origin, from an unsampled location to a discrete reservoir, such as human chronic infections (or even a network of chronic human infections) and/or animal reservoirs, potentially contributing to further evolution and dispersal of the virus, although, currently, the data do not provide any definitive evidence in any direction. We are actively investigating the potential of a yet unidentified animal reservoir in the region. To date, the only reverse zoonoses cases reported from the African region were in African lions and a puma in a private zoo in Johannesburg, South Africa^19^. Although these are unlikely species to play a role in the emergence of new variants, it is a reminder of the susceptibility of certain wildlife species to infections from humans. After the emergence of Omicron, the World Organisation for Animal Health released a statement calling for enhanced surveillance in animals to identify the origin of new variants^20^. Further genomic sampling and evolutionary investigation will, thus, be required to explain the origin of Omicron lineages.

In conclusion, we have identified two new Omicron lineages (BA.4 and BA.5), which are associated with a resurgence in infections in South Africa approximately 4 months on from the start of the Omicron wave. This once again highlights the importance of continued global genomic surveillance and variant analysis to act as an early warning system, giving countries time to prepare and mitigate the public health effect of emerging variants.

## Methods

### Epidemiological dynamics

We analyzed daily cases of SARS-CoV-2 in South Africa up to 29 May 2022 from publicly released data provided by the National Department of Health and the National Institute for Communicable Diseases. This was accessible through the repository of the Data Science for Social Impact Research Group at the University of Pretoria (https://github.com/dsfsi/covid19za)^21,22^. The National Department of Health releases daily updates on the number of confirmed new cases, deaths and recoveries, with a breakdown by province.

### Sampling of SARS-CoV-2

As part of the Network for Genomics Surveillance in South Africa (NGS-SA)^23^, seven sequencing hubs receive randomly selected samples for sequencing every week according to approved protocols at each site. These samples include remnant nucleic acid extracts or remnant nasopharyngeal and oropharyngeal swab samples from routine diagnostic SARS-CoV-2 PCR testing from public and private laboratories in South Africa. We analyzed SARS-CoV-2 genomes generated from samples collected between 1 November 2021 and 19 May 2022.

### Ethics statement

The genomic surveillance in South Africa was approved by the University of KwaZulu-Natal Biomedical Research Ethics Committee (BREC/00001510/2020), the University of the Witwatersrand Human Research Ethics Committee (HREC) (M180832), Stellenbosch University HREC (N20/04/008_COVID-19), the University of Cape Town HREC (383/2020), the University of Pretoria HREC (H101/17) and the University of the Free State Health Sciences Research Ethics Committee (UFS-HSD2020/1860/2710). The genomic sequencing in Botswana was conducted as part of the national vaccine roll-out plan and was approved by the Health Research and Development Committee (Health Research Ethics body, HRDC00948 and HRDC00904). Individual participant consent was not required for the genomic surveillance. This requirement was waived by the research ethics committees.

### Whole-genome sequencing and genome assembly

RNA was extracted on an automated chemagic 360 instrument, using the CMG-1049 kit (PerkinElmer). The RNA was stored at −80 °C before use. Libraries for whole-genome sequencing were prepared using either the Oxford Nanopore Midnight protocol with rapid barcoding or the Illumina COVIDseq Assay.

### Illumina Miseq/NextSeq

For the Illumina COVIDseq assay, the libraries were prepared according to the manufacturer’s protocol. In brief, amplicons were tagmented, followed by indexing using Nextera UD Indexes Set A. Sequencing libraries were pooled, normalized to 4 nM and denatured with 0.2 N sodium acetate. An 8 pM sample library was spiked with 1% PhiX (PhiX Sequencing Control v3 adaptor-ligated library used as a control). We sequenced libraries using the 500-cycle version 2 MiSeq Reagent Kit on the Illumina MiSeq instrument. On the Illumina NextSeq 550 instrument, sequencing was performed using the Illumina COVIDSeq protocol, an amplicon-based next-generation sequencing approach. The first-strand synthesis was performed using random hexamer primers from Illumina, and the synthesized cDNA underwent two separate multiplex PCR reactions. The pooled PCR amplified products were processed for tagmentation and adapter ligation using IDT for Illumina Nextera UD Indexes. Further enrichment and clean-up was performed according to protocols provided by the manufacturer (Illumina). Pooled samples were quantified using the Qubit 3.0 or 4.0 fluorometer (Invitrogen) and the Qubit dsDNA High Sensitivity assay kit according to manufacturer instructions. The fragment sizes were analyzed using TapeStation 4200 (Invitrogen). The pooled libraries were further normalized to 4 nM concentration, and 25 μl of each normalized pool containing unique index adapter sets was combined into a new tube. The final library pool was denatured and neutralized with 0.2 N sodium hydroxide and 200 mM Tris-HCl (pH 7), respectively. Sample library (1.5 pM) was spiked with 2% PhiX. Libraries were loaded onto a 300-cycle NextSeq 500/550 High Output Kit version 2 and run on the Illumina NextSeq 550 instrument.

### Midnight protocol

For Oxford Nanopore sequencing, the Midnight primer kit was used as described previously^1^. cDNA synthesis was performed on the extracted RNA using the LunaScript RT mastermix (New England Biolabs), followed by gene-specific multiplex PCR using the Midnight primer pools, which produce 1,200-bp amplicons that overlap to cover the 30-kb SARS-CoV-2 genome. Amplicons from each pool were pooled and used neat for barcoding with the Oxford Nanopore Rapid Barcoding Kit according to the manufacturer’s protocol. Barcoded samples were pooled and bead-purified. After the bead clean-up, the library was loaded on a prepared R9.4.1 flow cell. A GridION X5 or MinION sequencing run was initiated using MinKNOW software with the base-call setting switched off.

### Ion Torrent Genexus Integrated Sequencer methodology for rapid whole-genome sequencing of SARS-CoV-2

Viral RNA was extracted using the MagNA Pure 96 DNA and Viral Nucleic Acid Kit on the automated MagNA Pure 96 system (Roche Diagnostics) according to the manufacturer’s instructions. Extracts were then screened by qPCR to acquire the mean cycle threshold (Ct) values for the SARS-CoV-2 N and ORF1ab genes using the TaqMan 2019-nCoV Assay Kit version 1 (Thermo Fisher Scientific) on the ViiA7 Real-Time PCR System (Thermo Fisher Scientific) according to the manufacturer’s instructions. Extracts were sorted into batches of *n* = 8 within a Ct range difference of 5 for a maximum of two batches per run. Extracts with fewer than 200 copies were sequenced using the low-viral-titer protocol. Next-generation sequencing was performed using the Ion AmpliSeq SARS-CoV-2 Research Panel on the Ion Torrent Genexus Integrated Sequencer (Thermo Fisher Scientific), which combines automated cDNA synthesis, library preparation, templating preparation and sequencing within 24 hours. The Ion AmpliSeq SARS-CoV-2 Research Panel consists of two primer pools targeting 237 amplicons tiled across the SARS-CoV-2 genome providing >99% coverage of the SARS-CoV-2 genome (~30 kb) and an additional five primer pairs targeting human expression controls. The SARS-CoV-2 amplicons range from 125 bp to 275 bp in length. TRINITY was used for de novo assembly, and the Iterative Refinement Meta-Assembler (IRMA) was used for genome-assisted assembly as well as FastQC for quality checks.

### Genome assembly

We assembled paired-end and Nanopore.fastq reads using Genome Detective version 1.132 (https://www.genomedetective.com), which was updated for the accurate assembly and variant calling of tiled primer amplicon Illumina or Oxford Nanopore reads, and the Coronavirus Typing Tool. For Illumina assembly, the GATK HaploTypeCaller–min-pruning 0 argument was added to increase mutation calling sensitivity near sequencing gaps. For Nanopore, low-coverage regions with poor alignment quality (<85% variant homogeneity) near sequencing/amplicon ends were masked to be robust against primer drop-out experienced in the spike gene, and the sensitivity for detecting short inserts using a region-local global alignment of reads was increased. We also used the wf_artic (ARTIC SARS-CoV-2) pipeline as built using the Nextflow workflow framework. In some instances, mutations were confirmed visually with .bam files using Geneious version 2020.1.2 (Biomatters). The reference genome used throughout the assembly process was NC_045512.2 (numbering equivalent to MN908947.3).

Raw reads from the Illumina COVIDSeq protocol were assembled using the Exatype NGS SARS-CoV-2 pipeline version 1.6.1 (https://sars-cov-2.exatype.com/). This pipeline performs quality control on reads and then maps the reads to a reference using Examap. The reference genome used throughout the assembly process was NC_045512.2 (accession number MN908947.3).

Several of the initial Ion Torrent genomes contained several frameshifts, which caused unknown variant calls. Manual inspection revealed that these were probably sequencing errors resulting in mis-assembled regions (probably due to the known error profile of Ion Torrent sequencers). To resolve this, the raw reads from the Ion Torrent platform were assembled using the SARS-CoV-2 RECoVERY (Reconstruction of Coronavirus Genomes & Rapid Analysis) pipeline implemented in the Galaxy instance ARIES (https://aries.iss.it). This pipeline fixed the observed frameshifts, confirming that they were artifacts of mis-assembly; this subsequently resolved the variant calls. The Exatype and RECoVERY pipelines each produce a consensus sequence for each sample. These consensus sequences were manually inspected and polished using AliView version 1.27 (http://ormbunkar.se/aliview/).

All of the sequences passing internal quality control were deposited in GISAID (https://www.gisaid.org/), and the GISAID accession identifiers are included as part of Extended Data Table 1.

### Phylogenetic analysis

We initially analyzed genomes from South Africa against the global reference dataset using a custom pipeline based on a local version of NextStrain (https://github.com/nextstrain/ncov)^24^. The pipeline contains several Python scripts that manage the analysis workflow. It performs an alignment of genomes in NextAlign^25^, phylogenetic tree inference in IQ-TREE version 1.6.9 (ref. ^26^), tree dating and ancestral state construction and annotation (https://github.com/nextstrain/ncov).

The initial phylogenetic analysis enabled us to identify clusters corresponding to the BA.4 (*n* = 120) and BA.5 (*n* = 51) lineages. We extracted these clusters and constructed a preliminary maximum likelihood tree with a subset of BA.2 sequences (*n* = 52) in IQ-TREE. We inspected this maximum likelihood tree in TempEst version 1.5.3 (ref. ^27^) for the presence of a temporal or molecular clock signal. Linear regression of root-to-tip genetic distances against sampling dates indicated that the SARS-CoV-2 sequences evolved in a relatively strong clock-like manner (correlation coefficient = 0.6, R^2^ = 0.4).

Given that the estimation of time of the most recent common ancestor (TMRCA) and dispersal dynamics of the sampled viruses is best achieved using Bayesian phylogenetic methods, we then estimated time-calibrated phylogenies using the Bayesian software package BEAST version 1.10.4 (ref. ^28^). For this analysis, we used the strict molecular clock model, the HKY + I + G nucleotide substitution model and the exponential growth coalescent model^29^. We computed Markov chain Monte Carlo (MCMC) in duplicate runs of 20 million states each, sampling every 2,000 steps. Convergence of MCMC chains was checked using Tracer version 1.7.1 (ref. ^30^). Maximum clade credibility trees were summarized from the MCMC samples using TreeAnnotator after discarding 10% as burn-in. The phylogenetic trees were visualized using ggplot and ggtree^31,32^.

### Phylogeographic analysis

To model phylogenetic diffusion of the new cluster across the country, we used a flexible relaxed random walk diffusion model that accommodates branch-specific variation in rates of dispersal with a Cauchy distribution^33^. For each sequence, latitude and longitude were attributed to the most precise district or provincial information available and linked to the diagnostic sample.

As described in ‘Phylogenetic analysis’, MCMC chains were run in duplicate for 10 million generations and sampled every 1,000 steps, with convergence assessed using Tracer version 1.7.1. Maximum clade credibility trees were summarized using TreeAnnotator after discarding 10% as burn-in. We used the R package seraphim^34^ to extract and map spatiotemporal information embedded in posterior trees.

### Lineage classification

We used a previously proposed dynamic lineage classification method^35^ from the ‘Phylogenetic Assignment of Named Global Outbreak Lineages’ (pangolin) software suite version 4.0.6 with the –Usher option (https://github.com/cov-lineages/pangolin)^36^. This is aimed at identifying the most epidemiologically important lineages of SARS-CoV-2 at the time of analysis, enabling researchers to monitor the epidemic in a particular geographic region. A lineage is a linear chain of viruses in a phylogenetic tree showing connection from the ancestor to the last descendant. Variant refers to a genetically distinct virus with different mutations to other viruses.

### Selection analysis

To identify which (if any) of the observed mutations in the spike protein was most likely to increase viral fitness, we used the natural selection analysis of SARS-CoV-2 pipeline (https://observablehq.com/@spond/revised-sars-cov-2-analytics-page). This pipeline examines the entire global SARS-CoV-2 nucleotide sequence dataset for evidence of (1) polymorphisms having arisen in multiple epidemiologically unlinked lineages that have statistical support for non-neutral evolution (mixed-effects model of evolution)^37^; (2) sites at which these polymorphisms have support for a greater-than-expected ratio of non-synonymous-to-synonymous nucleotide substitution rates on internal branches of the phylogenetic tree (fixed-effects likelihood)^38^; and (3) whether these polymorphisms have increased in frequency in the regions of the world in which they have occurred.

### Estimating transmission advantage

We analyzed 15,225 SARS-CoV-2 sequences from South Africa generated in this study and uploaded to GISAID with sample collection dates from 1 November 2021 to 19 May 2022 (ref. ^39^). We used a multinomial logistic regression model to estimate the growth advantage of BA.4 and BA.5 over the other Omicron lineages^40,41^. We fitted the model using the multinom function of the nnet package and estimated the growth advantage using the package emmeans in R^42^.

### SGTF monitoring

SGTF monitoring was performed through analyzing SARS-CoV-2 laboratory test results from nasopharyngeal specimens received from the public health sector and referred for PCR testing undertaken by the National Health Laboratory Service (NHLS) in South Africa. The NHLS has a single laboratory information system connecting laboratory testing platforms to a corporate data warehouse, where data can be mined in near real time. The TaqPath COVID-19 assay (Thermo Fisher Scientific) accounts for around 20% of NHLS PCR tests performed, with around half of those performed in Gauteng. The TaqPath assay targets three gene regions, ORF1ab, N and S, with the lack of probe fluorescence of the latter culminating in SGTF. In Fig. 2a, we analyzed and plotted the weekly proportion of positive TaqPath tests with SGTF (defined as samples with non-detectable S-gene target and either N or ORF1ab gene positive with Ct value <30).

### Validation of S-gene target status as proxy for BA.4 and BA.5

Using a subset of unselected samples submitted to the KRISP sequencing laboratory, we compared the S-gene target status to the genome lineage assignment. In brief, RNA was extracted from nasopharyngeal swabs in viral transport media using the CMG-1033-S kit (chemagen, PerkinElmer). Then, 10 µl of purified RNA was amplified using the TaqPath COVID-19 CE-IVD RT–PCR Kit (Thermo Fisher Scientific) and analyzed with Design & Analysis software version 2.4. SGTF was denoted by lack of amplification of the S-gene target, with successful amplification of both the remaining ORF1ab and N-gene targets (Ct ≤ 30).

### Statistics

No statistical method was used to predetermine sample size. Data exclusion, randomization and blinding to allocation during experiments and outcome assessment were not applicable to this study.

### Reporting Summary

Further information on research design is available in the Nature Research Reporting Summary linked to this article.

## Online content

Any methods, additional references, Nature Research reporting summaries, source data, extended data, supplementary information, acknowledgements, peer review information; details of author contributions and competing interests; and statements of data and code availability are available at 10.1038/s41591-022-01911-2.

### Supplementary information

Reporting Summary

Supplementary Table 1 GISAID acknowledgement table

## Figures and Tables

**Extended Data Table 1:**
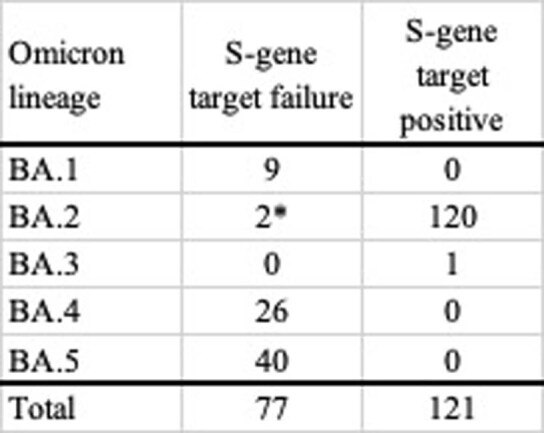
S-gene target status (TaqPath COVID-19 qPCR assay) for 198 samples sequenced by the KRISP laboratory. *One BA.2 sequence had the 69–70 deletion, and the other BA.2 sequence had large gaps in coverage of the spike gene region

**Extended Data Table 2:**
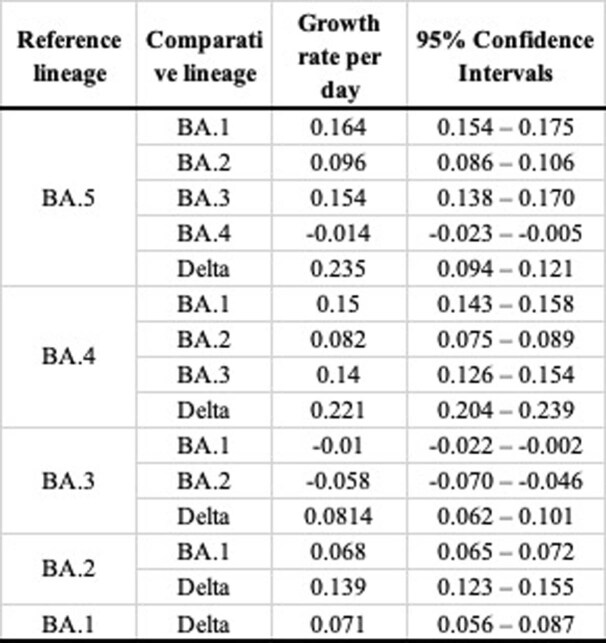
Comparison of daily growth rates of all Omicron lineages and Delta. Rates were estimated with multinomial logistic regression models based on South African SARS-CoV-2 genomic data spanning the period of 1 November 2021 to 19 May 2022. Negative values indicate the comparative lineage to have a growth advantage over the reference lineage, whereas a positive value indicates the reference lineage to have a growth rate advantage over the lineage of comparison

## Data Availability

All of the SARS-CoV-2 genomes generated and presented in this article are publicly accessible through the GISAID platform (https://www.gisaid.org/). The GISAID accession identifiers of the sequences analyzed in this study are provided as part of Supplementary Table 1. Other raw data for this study are provided as a supplementary dataset at https://github.com/krisp-kwazulu-natal/SARSCoV2_South_Africa_Omicron_BA4_BA5. The reference SARS-CoV-2 genome (MN908947.3) was downloaded from the National Center for Biotechnology Information database (https://www.ncbi.nlm.nih.gov/).

## Code availability

All custom scripts to reproduce the analyses and figures presented in this article are available at https://github.com/krisp-kwazulu-natal/SARSCoV2_South_Africa_Omicron_BA4_BA5.
