# Supplementary material for: Emergence of SARS-CoV-2 Omicron lineages BA.4 and BA.5 in South Africa
Source: Nat Med. 2022 Jun 27;28(9):1785–90. doi: 10.1038/s41591-022-01911-2 (PMC9499863; doi:10.1038/s41591-022-01911-2)
Supplement: Supplementary file 1 — Reporting Summary [file 41591_2022_1911_MOESM1_ESM.pdf]

nature portfolio

Corresponding author(s): Tulio de Oliveira

Last updated by author(s): Jun 18, 2022

Reporting Summary

Nature Portfolio wishes to improve the reproducibility of the work that we publish. This form provides structure for consistency and transparency in reporting. For further information on Nature Portfolio policies, see our Editorial Policies and the Editorial Policy Checklist.

Statistics

For all statistical analyses, confirm that the following items are present in the figure legend, table legend, main text, or Methods section.

n/a	Confirmed	
☐	☒	The exact sample size (n) for each experimental group/condition, given as a discrete number and unit of measurement
☐	☒	A statement on whether measurements were taken from distinct samples or whether the same sample was measured repeatedly
☒	☐	The statistical test(s) used AND whether they are one- or two-sided<br>*Only common tests should be described solely by name; describe more complex techniques in the Methods section.*
☒	☐	A description of all covariates tested
☐	☒	A description of any assumptions or corrections, such as tests of normality and adjustment for multiple comparisons
☐	☒	A full description of the statistical parameters including central tendency (e.g. means) or other basic estimates (e.g. regression coefficient) AND variation (e.g. standard deviation) or associated estimates of uncertainty (e.g. confidence intervals)
☒	☐	For null hypothesis testing, the test statistic (e.g. F, t, r) with confidence intervals, effect sizes, degrees of freedom and P value noted<br>*Give P values as exact values whenever suitable.*
☐	☒	For Bayesian analysis, information on the choice of priors and Markov chain Monte Carlo settings
☐	☒	For hierarchical and complex designs, identification of the appropriate level for tests and full reporting of outcomes
☒	☐	Estimates of effect sizes (e.g. Cohen's d, Pearson's r), indicating how they were calculated

Our web collection on statistics for biologists contains articles on many of the points above.

Software and code

Policy information about availability of computer code

Data collection	No software was used
Data analysis	Base-calling for GridIon sequencing was performed on MinKNOW software v21.6. Genome assembly was performed with Genome Detective online tool version 1.132 or Exatype NGS SARS-CoV-2 pipeline v1.6.1 or SARSCoV2 RECoVERY (REconstruction of COronaVirus gEnomes & Rapid analYsis) pipeline implemented in the Galaxy instance ARIES (https://aries.iss.it) and validated with Geneious software v.2020.1.2, IG Viewer or Aliview v1.27. Phylogenetic analysis was performed using Nextalign, IQ-Tree V1.6.9, TempEst v.1.5.3, BEASTv.1.10.4, BEAST2 v2.5.2, and Tracer v.1.7.1. Selection analyses were performed using HyPhy v2.5.33 through the RASCL pipeline. Lineage classification was performed using the PANGO software suite v4.0.6. R packages used for data analysis included ggplot, ggtree, seraphim. Custom codes are all available at: https://github.com/krisp-kwazulu-natal/SARSCoV2_South_Africa_Omicron_BA4_BA5.

For manuscripts utilizing custom algorithms or software that are central to the research but not yet described in published literature, software must be made available to editors and reviewers. We strongly encourage code deposition in a community repository (e.g. GitHub). See the Nature Portfolio guidelines for submitting code & software for further information.

Data

Policy information about availability of data

All manuscripts must include a data availability statement. This statement should provide the following information, where applicable:

- Accession codes, unique identifiers, or web links for publicly available datasets
- A description of any restrictions on data availability
- For clinical datasets or third party data, please ensure that the statement adheres to our policy

All of the SARS-CoV-2 genomes generated and presented in this manuscript are publicly accessible through the GISAID platform (https://www.gisaid.org/). The GISAID accession identifiers of the sequences analysed in this study are provided as part of Supplementary Table S1. Other raw data for this study are provided as a supplementary dataset at https://github.com/krisp-kwazulu-natal/SARSCoV2_South_Africa_Omicron_BA4_BA5. The reference SARS-CoV-2 genome (MN908947.3) was downloaded from the NCBI database (https://www.ncbi.nlm.nih.gov/).

Field-specific reporting

Please select the one below that is the best fit for your research. If you are not sure, read the appropriate sections before making your selection.

☒ Life sciences ☐ Behavioural & social sciences ☐ Ecological, evolutionary & environmental sciences

For a reference copy of the document with all sections, see nature.com/documents/nr-reporting-summary-flat.pdf

Life sciences study design

All studies must disclose on these points even when the disclosure is negative.

Sample size	No sample size calculation was performed; rather all genomic data available at the time of writing for the newly emerged BA.4 and BA.5 Omicron lineages was considered to ensure most accurate analysis and results in a timely manner. At the time of writing, 120 and 51 good quality sequences of the BA.4 and BA.5 SARS-CoV-2 lineages had been produced by the NGS-SA. We believe this was a sufficient sample size as the genomes spanned 7 of the 9 provinces of South Africa, including from multiple districts. For phylogentic analysis, this was analyzed against a representative set of 52 BA.2 SARS-CoV-2 genotypes.
Data exclusions	For phylogenetic analysis and time-calibrated BEAST analysis, genomes were excluded if they presented <90% coverage against the reference AND/OR have sequencing quality problem - e.g. gaps in key regions of the spike protein that causes spurious clustering.
Replication	Reproducibility were performed for bayesian MCMC phylogenetic tree reconstructions. We computed MCMC (Markov chain Monte Carlo) triplicate runs of 20 million states each, sampling every 2000 steps for the Omicron dataset. All attempts at replication were successful and the MCC tree for the BA.4 and BA.5 cluster was of high support.
Randomization	Experimental groups consisted of weekly batches of residual patient nasopharyngeal swabs selected for sequencing to determine the progression of weekly lineage prevalence as part of surveillance. Samples for weekly SARS-CoV-2 sequencing in South Africa and Botswana were selected at random from all relevant divisions in each country, without any clinical or geographical bias. Generally, part of the Network for Genomic Surveillance in South Africa (NGS-SA), seven sequencing hubs receive randomly selected samples for sequencing every week according to approved protocols at each site. Randomization of participants into experimental groups was not relevant to this study as experimental groups are determined by genomic viral classification into SARS-CoV-2 variants or lineages.
Blinding	Geographical blinding of data was not necessary for the study as it involves phylogeographical analysis. Other types of blinding were also not necessary as this was not a cohort study. Blinding of group assignment or outcome assessment were not applicable to this study as groups must be precisely assigned by genomic classification and outcomes need to be assessed in context of assigned genomic variant or lineages groups.

Reporting for specific materials, systems and methods

We require information from authors about some types of materials, experimental systems and methods used in many studies. Here, indicate whether each material, system or method listed is relevant to your study. If you are not sure if a list item applies to your research, read the appropriate section before selecting a response.

Materials & experimental systems

n/a	Involved in the study
☒	☐ Antibodies
☒	☐ Eukaryotic cell lines
☒	☐ Palaeontology and archaeology
☒	☐ Animals and other organisms
☐	☒ Human research participants
☒	☐ Clinical data
☒	☐ Dual use research of concern

Methods

n/a	Involved in the study
☒	☐ ChIP-seq
☒	☐ Flow cytometry
☒	☐ MRI-based neuroimaging

Human research participants

Policy information about studies involving human research participants

Population characteristics	We obtained samples consisting of remnant nucleic acid extracts or remnant nasopharyngeal and oropharyngeal swab samples from routine diagnostic SARS-CoV-2 PCR testing from public and private laboratories in South Africa. The Omicron genomes in this study came from patients of ages 0-82, with an approximately equal distribution of males and females, for which the Omicron genotype was confirmed by sequencing.
Recruitment	As part of the Network for Genomic Surveillance in South Africa (NGS-SA), seven sequencing hubs receive randomly selected samples for sequencing every week according to approved protocols at each site. One bias that may be present is the ability to sequence only from the pool of patients that seek testing and that receive a positive PCR test.
Ethics oversight	The genomic surveillance in South Africa was approved by the University of KwaZulu–Natal Biomedical Research Ethics Committee (BREC/00001510/2020), the University of the Witwatersrand Human Research Ethics Committee (HREC) (M180832), Stellenbosch University HREC (N20/04/008_COVID-19), University of Cape Town HREC (383/2020), University of Pretoria HREC (H101/17) and the University of the Free State Health Sciences Research Ethics Committee (UFS-HSD2020/1860/2710). The genomic sequencing in Botswana was conducted as part of the national vaccine roll-out plan and was approved by the Health Research and Development Committee (Health Research Ethics body, HRDC#00948 and HRDC#00904). Individual participant consent was not required for the genomic surveillance. This requirement was waived by the Research Ethics Committees.

Note that full information on the approval of the study protocol must also be provided in the manuscript.

March 2021
